# Interrelationships among Fatty Acid Composition, Staphyloxanthin Content, Fluidity, and Carbon Flow in the *Staphylococcus aureus* Membrane

**DOI:** 10.3390/molecules23051201

**Published:** 2018-05-17

**Authors:** Kiran B. Tiwari, Craig Gatto, Brian J. Wilkinson

**Affiliations:** School of Biological Sciences, Illinois State University, Normal, IL 61761, USA; kbtiwar@ilstu.edu (K.B.T.); cgatto@ilstu.edu (C.G.)

**Keywords:** membrane fatty acids composition, *Staphylococcus aureus*, staphyloxanthin, membrane fluidity, metabolic regulation

## Abstract

Fatty acids play a major role in determining membrane biophysical properties. *Staphylococcus aureus* produces branched-chain fatty acids (BCFAs) and straight-chain saturated fatty acids (SCSFAs), and can directly incorporate exogenous SCSFAs and straight-chain unsaturated fatty acids (SCUFAs). Many *S. aureus* strains produce the triterpenoid pigment staphyloxanthin, and the balance of BCFAs, SCSFAs and staphyloxanthin determines membrane fluidity. Here, we investigated the relationship of fatty acid and carotenoid production in *S. aureus* using a pigmented strain (Pig1), its carotenoid-deficient mutant (Pig1Δ*crtM*) and the naturally non-pigmented *Staphylococcus argenteus* that lacks carotenoid biosynthesis genes and is closely related to *S. aureus*. Fatty acid compositions in all strains were similar under a given culture condition indicating that staphyloxanthin does not influence fatty acid composition. Strain Pig1 had decreased membrane fluidity as measured by fluorescence anisotropy compared to the other strains under all conditions indicating that staphyloxanthin helps maintain membrane rigidity. We could find no evidence for correlation of expression of *crtM* and fatty acid biosynthesis genes. Supplementation of medium with glucose increased SCSFA production and decreased BCFA and staphyloxanthin production, whereas acetate-supplementation also decreased BCFAs but increased staphyloxanthin production. We believe that staphyloxanthin levels are influenced more through metabolic regulation than responding to fatty acids incorporated into the membrane.

## 1. Introduction

It is generally accepted that the fatty acid composition of membrane phospholipids have a major impact on determining membrane biophysical and, thereby, physiological properties [1]. *Staphylococcus aureus* is a Gram-positive bacterial pathogen that shows a significant growth environment-dependent plasticity in its membrane fatty acid composition [2]. The fatty acids of *S. aureus* are generally considered to be a mixture of saturated branched-chain fatty acids (BCFAs) and straight-chain fatty acids (SCSFAs) that are synthesized by fatty acid biosynthesis system II (FAS II). Some bacteriological media such as Mueller-Hinton broth (MHB) promote a high proportion of BCFAs, whereas others such as Tryptic Soy Broth (TSB) result in decreased BCFAs and increased SCSFAs. BCFAs fluidize the membrane and SCSFAs have the opposite effect [1,3,4,5]. The majority of BCFAs are anteiso- having a methyl group at the antepenultimate position while iso-fatty acids have a methyl group at the penultimate position. *S. aureus* lacks a fatty acid desaturase enzyme that converts saturated fatty acids into unsaturated products [2,6], and, hence, BCFAs play a critical role in maintaining membrane fluidity. However, when cells are grown ex vivo in serum straight-chain unsaturated fatty acids (SCUFAs) become a significant fraction of the total fatty acid composition [2], due to direct incorporation of exogenous preformed SCUFAs from serum into the phospholipid biosynthesis pathway [6]. SCUFAs are known to significantly increase membrane fluidity [1].

The membrane composition and properties of *S. aureus* are further complicated by the unique production of the triterpene staphyloxanthin carotenoid responsible for the typical golden color of *S. aureus* colonies. Staphyloxanthin is thought to be important in the life of *S. aureus* decreasing membrane fluidity [2,7,8,9,10,11,12,13], influencing membrane permeability [8], susceptibility to oxidative stress and neutrophil killing [14,15,16]. Thus staphyloxanthin biosynthesis has been explored as a potential drug target [16]. Fatty acid biosynthesis is one of the highly energy demanding biosynthetic pathways and acetyl-CoA is the common precursor for both fatty acid and staphyloxanthin synthesis [17]. Recently, we found a correlation between a high content of the membrane fluidizing fatty acids (BCFAs and SCUFAs) and an increase in staphyloxanthin content, possibly to counterbalance potential membrane hyperfluidity conferred by BCFAs or SCUFAs [2]. Similarly, a *S. aureus* mutant in *brnQ1* that was defective in transport of BCFA precursor amino acids had higher anteiso-fatty acids and increased pigmentation [18]. Here, we were interested in probing further the relationship between carotenoid and fatty acid production and membrane biophysical properties in pigmented (Pig1) and non-pigmented (Pig1Δ*crtM* and MSHR1132) staphylococcal strains. *S. aureus* Pig1Δ*crtM* is deleted in the *crtM* gene that encodes a key enzyme in staphyloxanthin production [14]. Strain MSHR1132 is a close relative of *S. aureus* that naturally lacks the carotenoid biosynthesis operon (*crtOPQMN*) that is known as *Staphylococcus argenteus* (the silver *Staphylococcus*, [19,20]. 

The level of staphyloxanthin production varies between strains and under different environmental conditions and the role of the pigment in the life of *S. aureus* remains somewhat enigmatic. The alternative sigma factor SigB is a positive regulator of expression of the *crtMNOPQ* operon [21], and direct or indirect effects on the expression and activity of SigB regulate pigment production in the bacterium. A cold-shock protein (CspA) [22] and an aeration sensing response regulator (AirR) [23] have been shown to positively affect SigB activity and increase pigmentation. Mutations and/or altered activities of some regulators (such as SarA, Agr, argR) and ClpP protease may also affect SigB expression altering pigmentation [17,24]. Also, a transfer-messenger antisense RNA has been reported that negatively regulates the *crtMN* transcript [25]. Additionally, a carbohydrate catabolite regulator (CcpE) has been shown to regulate pigment production without affecting *crtM* transcription [26,27]. On the other hand, addition of fatty acids or mevalonate in the growth medium [28], blocking polyprenyl synthetase [24] and inactivation of the tricarboxylic acid (TCA) cycle [17] have been reported to increase pigmentation. A *S. aureus* pyruvate dehydrogenase mutant defective in conversion of pyruvate to acetyl CoA had decreased staphyloxanthin production, whereas a branched-chain α-keto acid dehydrogenase mutant had decrease production of C4 and C5 iso and anteiso CoA precursors of BCFAs showed increased pigmentation [29]. 

The membrane lipid composition of *S. aureus* is complex due to the variable production and incorporation of BCFAs, SCSFAs and SCUFAs, as well as being overlain with the variable production of staphyloxanthin. In earlier work, we observed an association between increased content of membrane fluidizing BCFAs and SCUFAs and increased content of membrane rigidifying staphyloxanthin. In this work we probed this potential relationship further through studies of fatty acid composition, staphyloxanthin content, and membrane fluidity in a carotenoid-deficient *S. aureus* mutant and a naturally occurring staphylococcal species closely related to *S. aureus* that had lost the *crt* carotenoid biosynthesis operon. We found no evidence that carotenoid biosynthesis responded to membrane fatty acid composition. Carotenoid deficiency led to membranes with increased fluidity. However, medium carbon source influenced membrane fatty acid composition and staphyloxanthin content, with evidence that excess acetyl CoA led to increased staphyloxanthin production.

## 2. Results

### 2.1. The Fatty Acid Composition of S. aureus Pig1 Varies with the Growth Medium

To understand variations in fatty acid composition in a pigmented *S. aureus* strain grown in various media, we analyzed the fatty acid composition of TSB-, MHB- and serum-grown *S. aureus* Pig1 by fatty acid methyl-ester analysis. We observed nearly half each of SCSFAs (51%) and BCFAs (49%) in log phase cells grown in TSB (Table 1). However, MHB-grown cells had 81% BCFAs and the serum-grown cells had a significant proportion of SCUFAs (41%) (Table 1). Stationary phase cells grown in TSB and MHB had increased proportions of BCFAs (76% and 94% respectively) with a decrease in SCSFAs. However, cells grown in serum at stationary phase had similar proportion of the fatty acid types found in the log phase cells. Cells grown in serum at log phase had 30% oleic acid (18:1Δ9) and 8% gondoic acid (20:1Δ11) as major SCUFAs. Notably, stationary phase cells grown in serum had 12% linoleic acid (18:2Δ9, 12) besides oleic acid (17%), and gondoic acid (5%) as major SCUFAs (Supplementary Materials Table S2). In summary, fatty acid composition varied considerably with growth medium and growth phase.

### 2.2. The Carotenoid Content of Pig1 Varies with the Growth Medium

To understand the variations in cellular pigmentation in different growth media, staphyloxanthin content in Pig1 cells grown in TSB, MHB and serum was determined by the spectrophotometric method. Staphyloxanthin content was expressed as OD_465_ per mg dry wt. of cell pellets. Compared to staphyloxanthin content (0.017 ± 0.001) in the TSB-grown cells at log phase, the MHB and serum grown cells had higher pigmentation (0.051 ± 0.003 and 0.036 ± 0.003 respectively) (Table 2). However, cells grown to stationary phase in TSB had a higher staphyloxanthin content than those grown in other media. TSB- and MHB-grown cells were most pigmented after 12–24 h of growth whereas the serum-grown cells had similar pigmentation over the entire growth period (Table 2). In summary, pigmentation varied considerably with growth media and over the growth phases in a given medium, and, in general, the cells having a higher proportion of BCFAs had a higher staphyloxanthin content.

### 2.3. The Carotenoid-Deficient Strains Had Essentially Similar Proportions of Fatty Acids to the Pigmented Strains

**Figure 1:**
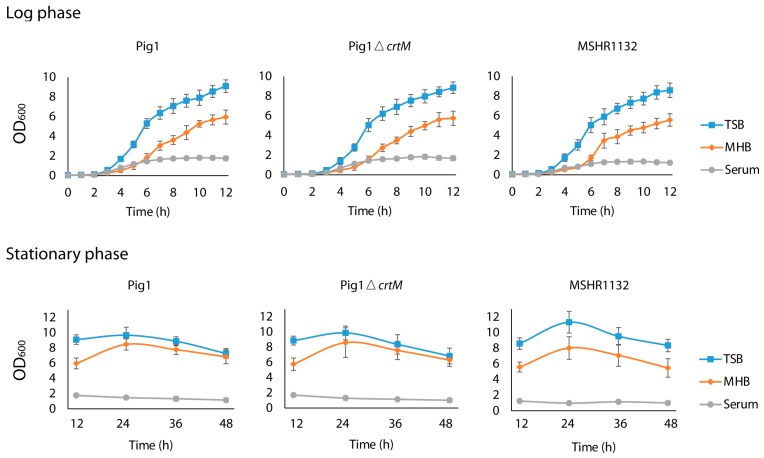
Staphyloxanthin production does not affect growth patterns of pigmented and non-pigmented *S. aureus* grown in various media. The strains were grown in TSB, MHB and serum and optical density (OD_600_) was measured at intervals. Error bars indicate standard deviation on the mean OD_600_ values determined from three independent growth experiments for each strain.

The lack of pigmentation had no effect in the overall growth patterns of *S. aureus* strains grown in various media (Figure 1). To understand whether the lack of pigmentation affects the fatty acid composition in *S. aureus* the fatty acid composition was determined in the TSB-, MHB and serum-grown carotenoid-deficient mutant (Pig1Δ*crtM*) and *S. argenteus*. The proportion of fatty acids of Pig1Δ*crtM* grown in different media at both log and stationary phases was very similar to those observed in the parent strain (Table 1 and Table 3). Strain MSHR1132 also had a similar fatty acid composition compared to the other two strains (Table 1, Table 3 and Table 4); one difference was the presence of linoleic acid (C18:2Δ9, 12) when grown in serum at both log (7%) and stationary (10%) phases whereas in the other serum-grown strains this fatty acid was present only at the stationary phase (Pig1, 12%; Pig1Δ*crtM*, 11%) (Supplementary Materials Table S2). Collectively, the overall similarities of the fatty acid composition in the *S. aureus* strains were unaffected by the loss of ability to produce staphyloxanthin.

### 2.4. Alteration in Fatty Acid Composition Significantly Affects Membrane Fluidity in Staphyloxanthin-Lacking S. aureus

**Figure 2:**
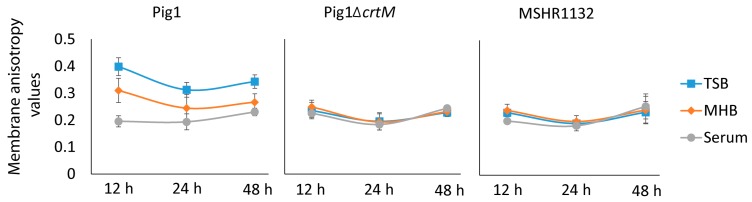
Staphyloxanthin in the pigmented *S. aureus* helps cells maintain membrane fluidity over stationary phase too. Membrane anisotropy values of the stationary phase cells were determined using a membrane intercalating fluorescent dye (DPH) as described for the log phase cells. Note that anisotropy values do not have unit. Error bars indicate standard deviation from mean anisotropy values obtained from at least three independent experiments.

To understand how the loss of staphyloxanthin affects membrane biophysical properties in *S. aureus* we determined membrane anisotropies of strains Pig1, Pig1Δ*crtM* and MHSR1132 grown in TSB, MHB and serum. A membrane with lower anisotropy value indicates higher fluidity. As shown in Table 5, the Pig1 cells grown in TSB and MHB at log phase had similar anisotropy values (0.315 ± 0.025 and 0.318 ± 0.035 respectively) while the cells grown in serum had significantly more fluid membranes (0.212 ± 0.017). The MHB-grown Pig1 cells had higher staphyloxanthin content as well as a higher proportion of membrane fluidizing BCFAs. On the other hand, the carotenoid lacking mutant (Pig1Δ*crtM*) had significantly higher membrane fluidity (TSB, 0.260 ± 0.033; MHB, 0.202 ± 0.032; serum, 0.159 ± 0.007) compared to its similarly grown pigmented parent strain. The MHB-grown carotenoid-deficient cells had significantly higher membrane fluidity than those grown in TSB. Additionally, the naturally carotenoid-lacking strain MSHR1132 (*S. argenteus*) had significantly higher membrane fluidities (TSB, 0.254 ± 0.024; MHB, 0.239 ± 0.010; serum, 0.135 ± 0.016) compared to the Pig1 strain. On the other hand, the Pig1 strain grown in TSB at stationary phase had higher staphyloxanthin content (see Table 2) and, thus, had correspondingly higher membrane rigidity (lower fluidity) than it did when grown in other media (Figure 2). The carotenoid-lacking strains grown in all media had highly fluid membranes (Figure 2). These observations also support the idea that staphyloxanthin content corresponds to increased membrane rigidity, and this relationship prevails over the entire growth period. In summary, staphyloxanthins play a major role in maintenance of membrane viscosity in *S. aureus* and alteration of fatty acid composition significantly affects membrane fluidity in the non-pigmenting strains.

### 2.5. Expression of the Genes for Staphyloxanthin and Fatty Acid Biosynthesis Do Not Appear to Be Co-Regulated

**Figure 3:**
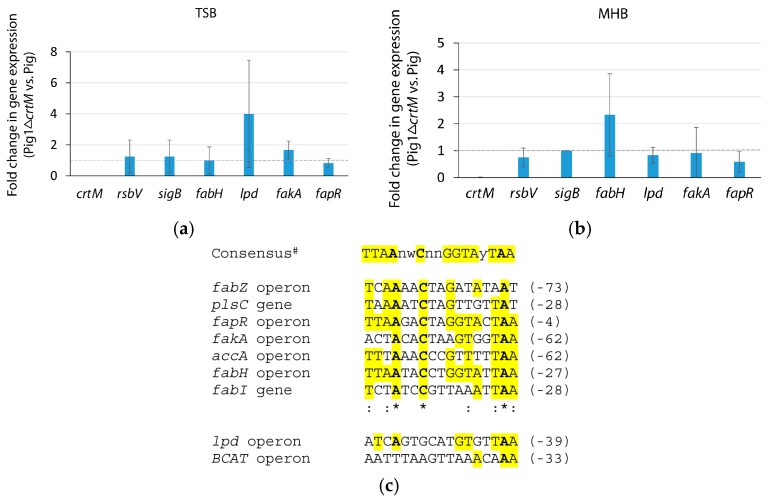
Transcription of fatty acid biosynthesis genes is typically unaffected by the loss of staphyloxanthin production in *S. aureus*. (**a**) Fold change in gene expression in Pig1Δ*crtM* compared to that in Pig1 grown in TSB; (**b**) Fold change in gene expression in Pig1Δ*crtM* compared to that in Pig1 grown in MHB; (**c**) FapR recognition sequence (operator sequence) in the gene or operon involved in fatty acid biosynthesis and regulation in *S. aureus* NCTC 8325. The transcriptional level of each gene was normalized to that of 16SrRNA. The dashed line in (**a**,**b**) indicates similar level of gene expression in the Pig1 pair strains. *fabZ* operon, *SAOUHSC_02337*–*02336*; *plsC*, *SAOUHSC_01837*; *fapR* operon, *SAOUHSC_01196–01199*; *fakA* operon, *SAOUHSC_01192–01193*; *accA* operon, *SAOUHSC_01809–01808*; *fabH* operon, *SAOUHSC_00920–00921*; *fabI*, *SAOUHSC_00947*; *lpd* operon, *SAOUHSC_01617–SAOUHSC_01615–01611*; *BCAT*, *SAOUHSC_00536–00537*. The number in parentheses after each operator sequence in (**c**) indicates the last nucleotide in the given sequence. ^#^ [30].

To understand whether there was cellular co-regulation of fatty acid biosynthesis and staphyloxanthin in *S. aureus* the level of expression of the genes encoding key enzymes involved in the biosynthesis and regulation of both pathways were analyzed by qRT-PCR assays. As expected, the transcript of the *crtM* gene in strain Pig1Δ*crtM* was barely detected (Figure 3A,B). Strain Pig1Δ*crtM* had a nearly similar level of expression of *rsbV* (positive regulator of *sigB*), *sigB* (regulator of *crtOPQMN* operon), *fabH* (initiation of fatty acid biosynthesis), *fakA* (fatty acid uptake) and *fapR* (negative regulator of fatty acid biosynthesis) genes when grown in TSB compared to that of its parent strain (Pig1) (Figure 3A). On the other hand, *rsbV*, *sigB* and *fakA* were similarly expressed while *fapR* was slightly downregulated and *fabH* was slightly upregulated in the MHB-grown mutant (Figure 3B). The relative differences in the *fapR* and *fabH* levels may indicate the MHB-grown mutant has slightly increased activity in fatty acid biosynthesis. However, in totality, the similar level of expression of most of the key genes for fatty acid and staphyloxanthin biosynthesis and their regulation in the pigmented and non-pigmented strains indicate that the pathways appear to be independently regulated. Notably, the expression of *lpd* (BCFA biosynthesis) in the TSB-grown mutant strain was markedly elevated (Figure 3A) while in MHB it was relatively unchanged (Figure 3B). In silico study of *S. aureus* NCTC8325 revealed that the operator sequence of the *lpd*-operon and *BCAT* gene (initial recruitment of branched-chain amino acids for BCFA biosynthesis) are unlikely be regulated by FapR (Figure 3C). Since the proportion of BCFAs in the staphyloxanthin-lacking mutant is similar to that of the pigmenting parent strain grown in a given medium the reason for the relative differences of the expression of *lpd* gene in TSB-grown strains is not clear.

### 2.6. Glucose and Acetate Affect Staphyloxanthin Production in S. aureus

**Figure 4:**
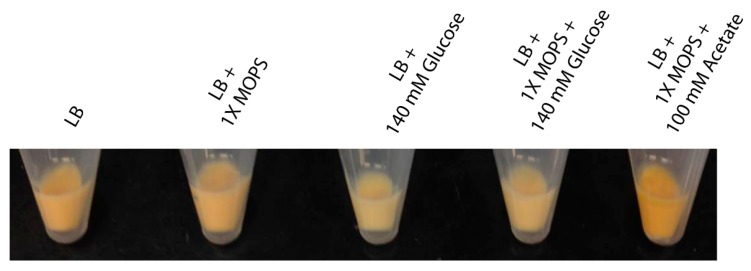
Visible differences in carotenoid content in the Pig1 cells grown in various LB broths. Strain Pig1 was grown for 12 h in LB broths with or without glucose or acetate. The cell pellets were collected and photographed.

TSB but not MHB contains free glucose, hence, the availability of glucose in the growth media might play a role in alteration of pigmentation. To investigate how glucose affects staphyloxanthin production in *S. aureus*, the change in pigmentation in strain Pig1 was studied by growing it in LB broth pH 7.2 (that lacks carbohydrates) supplemented with 140 mM glucose. Since glucose catabolism yields acetate [31], acetate (100 mM) containing LB broth was also included in this study. To avoid alteration of pH by acetate, acetate was supplemented in LB containing MOPS buffer (pH 7.2). Compared to the staphyloxanthin content in the plain LB-grown cells (log, 0.033 ± 0.007; stationary, 0.060 ± 0.007) a significantly reduced pigmentation was observed in LB broths to which glucose had been supplemented (0.021 ± 0.007 and 0.033 ± 0.004 respectively) (Table 6). Note that MOPS buffer had no effect on pigmentation. Exponentially growing cells in acetate-supplemented LB broth had a slightly higher staphyloxanthin content (0.038 ± 0.005) than in plain LB. A significantly higher pigmentation was observed in cells grown at stationary phase in acetate-containing LB (0.071 ± 0.004) (Table 6). The cell pellets obtained from stationary phase LB cultures had a visible difference in the depth of yellow color (Figure 4). In summary, the pigmentation in *S. aureus* decreases with the presence of glucose and increases with presence of acetate in the growth media.

### 2.7. Availability of Glucose and Acetate in the Growth Medium Lowers BCFAs and Increases SCSFAs in S. aureus

To understand how a carbon source affects fatty acid composition in a pigmented *S. aureus* strain strain Pig1 was grown in LB or LB supplemented with glucose or acetate and fatty acid composition was determined. Fatty acid analysis was done for the stationary phase cells that had significant differences in pigment production (see Table 6). As shown in Table 7, the LB-grown cells had 91% BCFAs and 9% SCSFAs. Addition of glucose in LB decreased the proportion of BCFAs to 63% and increased SCSFAs to 37%. Addition of acetate in the medium similarly decreased BCFA contents (63%) and increased SCSFAs (27%). These observations indicate that the carbon sources might be the major factors in determining the proportion of BCFAs and SCSFAs in *S. aureus*.

### 2.8. Glucose and Acetate also Affect Pigmentation in Other S. aureus Strains 

**Figure 5:**
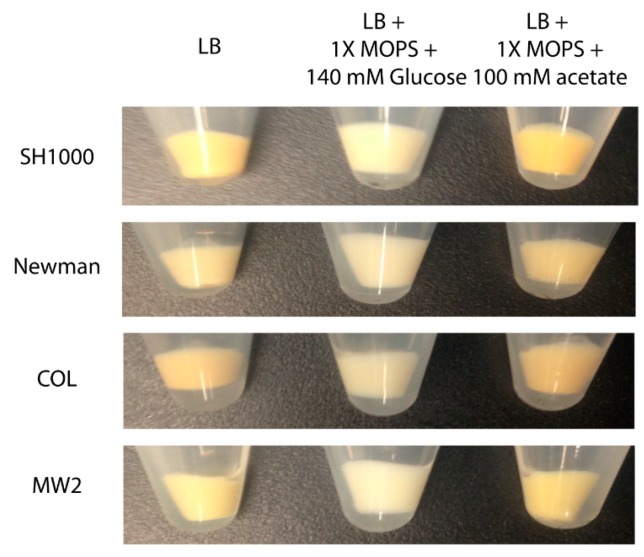
Pigmentation among various *S. aureus* strains grown in glucose or acetate added medium. Strains SH1000, Newman, COL and MW2 were grown for 12 h in LB broths without or with glucose or acetate. Cell pellets were collected and photographed.

To determine whether pigmentation in other *S. aureus* strains was affected by glucose or acetate, methicillin-susceptible (MSSA) strains (SH1000, laboratory derived MSSA; Newman, clinical isolate MSSA) and methicillin-resistant (MRSA) strains (COL, homogeneously resistant MRSA; MW2, heterogeneously resistant MRSA) were included in this study. The strains were grown in LB broth without or with glucose or acetate until stationary phase (12 h). Similar to the effects on pigmentation in strain Pig1, the cell pellets for all the strains (*viz*., SH1000, Newman, COL and MW2) obtained from the glucose-supplemented cultures had decreased pigmentation while those from acetate-supplemented cultures had increased pigmentation (Figure 5). This observation indicates that the effect of glucose and acetate on pigmentation extends to other pigmented *S. aureus* strains.

### 2.9. Acetate Consumption Determines the Extent of Pigment Production in S. aureus

**Figure 6:**
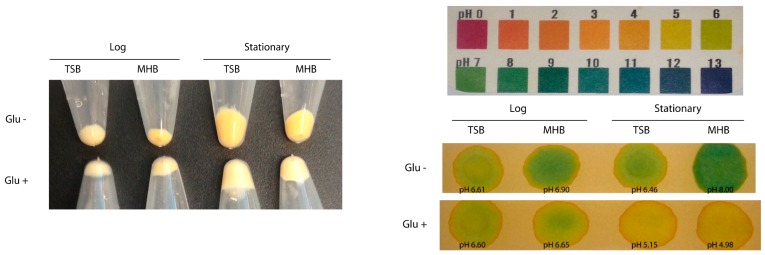
Effect of glucose in *S. aureus* Pig1 grown in TSB and MHB. (**a**) Pigmentation in cell pellets of strain Pig1 grown with or without glucose in the growth media. The strain was grown in plain TSB or MHB and glucose-added TSB or MHB at log phase (OD_600_ ~0.8) and at stationary (12 h; TSB, OD_600_ ~8.0; MHB, OD_600_ ~6.0). Cell pellets from 5 mL cultures were collected, washed, and photographed; (**b**) pH change in the culture supernatants. pH change in the corresponding culture supernatants was measured using a pH meter and pH paper (pHydrion Insta-Check, Micro Essential laboratory, B’klyn, NY). Five microliter of each supernatant was put on the pH paper. Upper panel shows the reference color for a given pH value.

*S. aureus* containing higher proportions of BCFAs also had higher levels of pigmentation in general (see Table 1 and Table 2). However, the cells having higher staphyloxanthin content did not always have a higher proportion of BCFAs (see Table 6 and Table 7 for LB-MOPS-Acetate grown cells; and Table 1 and Table 2 for 12 h-grown cells in TSB and MHB). These data suggest that staphyloxanthin and BCFAs levels are not always related and that glucose catabolism could be important in determining the relative amount of BCFAs and staphyloxanthin in the cell membrane. Exponentially-growing *S. aureus* consumes glucose converting it into acetyl-CoA that is secreted as acetate into the growth medium [31]. With the depletion of glucose during stationary phase the cells consume acetate from the growth medium, converting it into acetyl-CoA that feeds into the TCA cycle [31,32]. To demonstrate whether a change in pigmentation in *S. aureus* was due to consumption of acetate, acetate concentration in the culture supernatants in glucose-supplemented or plain TSB and MHB broths were determined. As shown in Table 8, *S. aureus* grown in glucose-supplemented MHB had higher acetate concentration in the supernatants collected from both log (5.17 ± 0.98 mM) and stationary (27.07 ± 1.63 mM) phase cells than those in plain MHB (3.09 ± 0.64 mM and 7.33 ± 1.02 mM respectively). Further, the cells grown in the glucose-supplemented MHB had correspondingly lower staphyloxanthin production (log, 0.035 ± 0.001; stationary, 0.031 ± 0.005) than cells in unsupplemented MHB (log, 0.051 ± 0.003; stationary, 0.086 ± 0.003) (Table 8). Similar relationships of lower acetate consumption and concomitantly lower pigmentation were observed in cells grown in glucose-supplemented TSB (Table 8). Similarly, the effect of glucose on acetate consumption was observed in the TSB-grown cells. Since TSB contains free glucose (14 mM), there was no obvious difference in acetate consumption and pigmentation in the glucose-supplemented broth during log phase growth (OD_600_ ~0.8). However, the effects of glucose-supplementation (140 mM) on acetate consumption and pigmentation were pronounced in the cells grown at stationary phase (OD_600_ ~8.0). The pigmentation in the cell pellets obtained from various growth conditions is shown in Figure 6A. Additionally, the corresponding pH in the culture supernatants is shown in Figure 6B. In summary, acetate consumption over the growth phases largely determines pigment production in *S. aureus*.

## 3. Discussion

Fatty acids in phospholipids play major roles in determining membrane biophysical properties. *S. aureus* synthesizes and alters the balance of membrane fluidizing BCFAs (mostly anteiso C15:0 and anteiso C17:0) and rigidifying SCSFAs under different growth conditions. The bacterium can take up SCUFAs from environment and incorporate them into phospholipids [2,6]. Many *S. aureus* strains produce a characteristic membrane component, staphyloxanthin, which is a triterpenoid pigment with an anteiso C15:0 at the C6-position of the glucose residue in glycosyl-4,4′-diaponeurosporenoate [10,12,13,33,34]. The pigment has been shown to provide rigidity in the membrane [2,7,8,9,10,11]. Here, we grew *S. aureus* strains Pig1 (pigmented), Pig1Δ*crtM* (non-pigmented), and *S. argenteus* (a close relative of *S. aureus*, non-pigmented) in various culture media and analyzed the relationship between fatty acid composition and pigmentation. We observed that production of staphyloxanthin does not influence the fatty acid composition but helps maintain membrane fluidity. Notably, fatty acid composition and pigmentation in the bacterium were markedly affected by supplementation of glucose or acetate in the growth medium. We believe that staphyloxanthin production and fatty acid composition are regulated metabolically. 

The relationship of fatty acid composition and staphyloxanthin production is complex. As observed previously [2] in two different strains of *S. aureus*, here we observed that *S. aureus* Pig1 grown in MHB at log phase had higher BCFAs and higher pigmentation than those grown in TSB. Further, serum-grown cells had a large proportion of SCUFAs and higher pigmentation than the TSB-grown cells. Additionally, we observed that the 12 h-grown cells in TSB and MHB had higher proportion of BCFAs and were more pigmented than the respective log phase cells indicating a direct relationship of BCFAs and pigmentation in *S. aureus*. However, despite having lower BCFAs, the TSB-grown cells at 12 h had markedly higher pigmentation than those grown in MHB. Further, despite having a substantial proportion of SCUFAs the serum-grown cells at 12 h had least pigmentation. Additionally, acetate-supplementation in culture broths increased pigmentation and decreased BCFAs in *S. aureus*. These observations indicate that there may not always be a direct relationship of fatty acid composition and pigmentation in *S. aureus*. We observed that the fatty acid composition in pigmented and non-pigmented strains were similar. Further, the genes for biosynthesis and regulation of fatty acids and staphyloxanthin in the strains were appear to be independently regulated. Taken together our observation suggest that the relationship of fatty acid composition and pigmentation in *S. aureus* is complex.

*S. aureus* acetate metabolism determines the extent of its pigment production. *S. aureus* continuously secretes acetate in glucose-containing medium and consumes acetate when glucose is exhausted [31]. We observed that supplementation of media with glucose decreased pigmentation while acetate-supplementation increased pigmentation. Further, glucose-supplementation increased acetate concentration in the culture supernatants even at stationary phase where the cells preferentially consume acetate otherwise. Since glucose catabolism yields acetyl-CoA and the excess acetyl-CoA is converted into acetate, which is continuously secreted out of cells [31], the secretion of the precursor molecule might reduce pigmentation in the cells in glucose-supplemented media. On the other hand, supplementation of acetate in the media might provide surplus acetyl CoA in the cells leading to higher pigment production. Hence, these observations indicate that acetate consumption directly correlates to pigmentation in *S. aureus*. 

**Figure 7:**
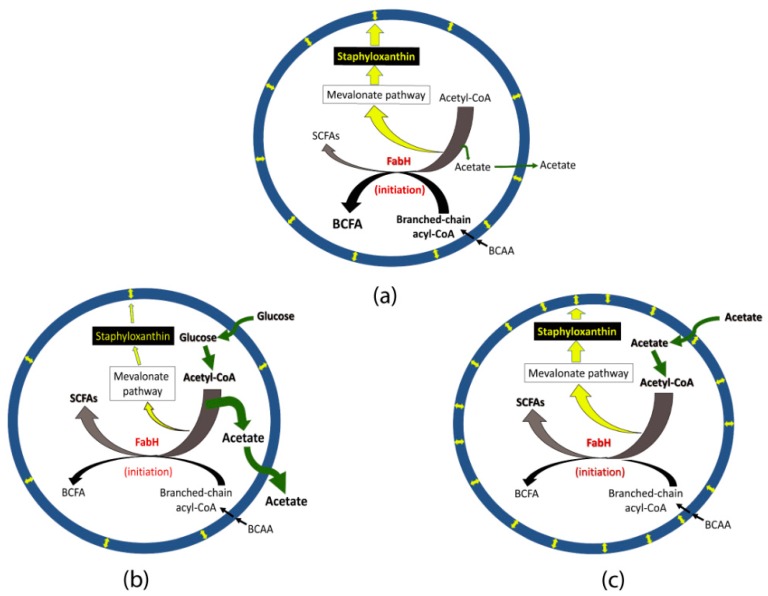
Proposed model on alterations in fatty acid composition and staphyloxanthin in *S. aureus*. Upper model (**a**) shows that higher proportion of BCFAs is achieved with higher affinity of FabH (initiation of fatty acid biosynthesis) for branched-chain acyl-CoA leaving abundant amounts of acetyl-CoA that can overflow towards staphyloxanthin production when *S. aureus* grown in glucose-lacking media such as LB or MHB. Lower left model (**b**) shows that glucose-supplementation in the growth medium causes production of excess acetyl-CoA molecules that are mostly converted into acetate and secreted outside. Note that the continuous supply of acetyl-CoA may favor an increased proportion of SCSFAs compared to cells grown without glucose-supplementation; Lower right model (**c**) shows that acetate-supplementation increases acetyl-CoA pool in the cytoplasm and, thus, increased the production of staphyloxanthin and SCSFAs. BCAA, branched-chain amino acids (valine, leucine, isoleucine). Note that acetyl-CoA is also used to make malonyl-CoA to be used in fatty acid elongation. The elongation process is the same for both SCSFA and BCFA synthesis utilizing malonyl-CoA biosynthesized from acetyl-CoA. The yellow double-arrowheads represent staphyloxanthin molecules in the membrane (thick blue circles).

Acetate metabolism may affect initiation of fatty acid biosynthesis leading to alteration in BCFA and staphyloxanthin production. As explained above, increased BCFAs is usually associated with increased pigmentation in glucose-lacking media (Figure 7a); however, an increased pigmentation may not result in increased BCFAs. This notion is supported by an observation that a *S. aureus pdh* mutant impaired in acetyl-CoA production had both significantly reduced staphyloxanthin and significantly higher BCFAs (80%; wild type, 50%) [29]. Similarly, a *bkd* mutant had significantly higher pigmentation but had significantly lower BCFAs (31%) [29]. Since, BCFA synthesis requires branched-chain acyl-CoA (precursors synthesized by the BKD complex) and fatty acid biosynthesis initiation enzyme (FabH) prefers branched-chain acyl-CoA to acetyl-CoA [35], the scarcity of acetyl-CoA in the *pdh* mutant might be a condition where the FabH enzyme synthesizes more BCFAs. On the other hand, a decreased synthesis of BCFAs in the *bkd* mutant might result in FabH enzyme utilizing more acetyl-CoA in the initiation step leading to increased SCSFAs than in the parent strain. Based on this assumption of acetyl-CoA consumption in a *bkd* mutant, a continuous production (cellular availability) of acetyl-CoA in glucose-supplemented broths might affect FabH activity leading to increased SCSFAs (Figure 7b). Similarly, a continuous uptake of acetate and subsequent availability of surplus acetyl-CoA could affect FabH activity leading to increased SCSFAs in the acetate-supplemented medium (Figure 7c). 

An increased flow of acetyl CoA causes a higher pigmentation in *S. aureus* Pig1. The notion of increased flow of acetyl-CoA towards the mevalonate pathway leading to increased pigmentation is corroborated by the findings of addition of fatty acids or mevalonate in the growth medium [28], blocking polyprenyl synthetase [24] and inactivation of the TCA cycle [17] increase pigmentation in *S. aureus*. Inactivation of the TCA cycle provides more acetyl-CoA in the cytoplasmic pool that can be used in the mevalonate pathway [17]. Notably, the mevalonate pathway also yields undecaprenyl phosphate that is required to synthesize peptidoglycan, teichoic acid, and capsule [36] and menaquinone. Interestingly, most (~90%) of the undecaprenyl phosphate is converted and stored as an inactive form, undecaprenol, which is recycled back to undecaprenyl phosphate when necessary [36,37]. Therefore, we believe that consumption of most of the surplus acetyl-CoA by the mevalonate pathway is committed to staphyloxanthin production.

On the other hand, a highly pigmented *bkd* mutant had an unaltered level of *crtM* transcript compared to the parent strain indicating an unaltered SigB (that regulates the *crt* operon) activity in the mutant [29]. Hence, an increased flow of acetyl-CoA towards the mevalonate pathway could be responsible for increased pigmentation in the *bkd* mutant [29]. In this study, we supplemented broths with glucose (140 mM) and acetate (100 mM) to alter the carbon flow in a clinical isolate Pig1. A decreased pigmentation with glucose-supplementation and an increased pigmentation with acetate-supplementation was observed in both log- and stationary-phase cells indicating that the alterations in pigmentation might not be influenced by SigB (the activity of which changes over growth cycle, [38,39,40]). Taken altogether, an increased flow of carbon to the mevalonate pathway might cause an increase in pigmentation in *S. aureus* Pig1 having higher proportion of BCFAs. 

## 4. Materials and Methods

*S. aureus* strains and growth media. A markedly yellow pigmented clinical *S. aureus* isolate (Pig1) and its non-pigmented *crtM*-knockout mutant (Pig1Δ*crtM*) [14], kindly supplied by Dr. George Liu, University of California San Diego, La Jolla, CA, and a naturally *crt*-operon lacking clinical *S. argenteus* strain (Deutsche Sammlung von Mikroorganismen und Zellkulturen (DSMZ), Leibniz-Institut, Germany [19,20]) were used in this study (Table 9). Other *S. aureus* strains used were SH1000 [41], Newman [42], COL [43] and MW2 [44] (Table 9). The growth media (culture to volume ratio of 1:5) used were Bacto^TM^ Tryptic soy broth (TSB; Becton, Dickinson and Company, Baltimore, MD, USA), Bacto^TM^ Mueller-Hinton broth (MHB; Becton, Dickinson and Company, MD), Difco^TM^ Luria-Bertani (LB, Miller; Becton, Dickinson and Company) medium and whole human serum (BioreclammationIVT, New York, NY, USA). Human serum was heated to 56 °C for 30 min before using.

Determination of growth patterns of *S. aureus* strains grown in TSB, MHB and human serum. Overnight grown strains Pig1, Pig1Δ*crtM* and MHSR1132 were inoculated into TSB, MHB and serum, grown for 48 h and growth was monitored by measuring turbidity (OD_600_) at intervals. 

Determination of fatty acid compositions in *S. aureus* strains grown in TSB, MHB and human serum. Strains Pig1, Pig1Δ*crtM* and MHSR1132 were grown to an optical density (OD_600_) of about 0.8 in TSB, MHB and serum, were harvested (3000× *g* for 5 min), washed with cold water, and the pellets were sent as frozen sample with overnight delivery for fatty acid methyl ester (FAME) analysis at MIDI, Inc. (Newark, Delaware, USA). At MIDI, fatty acids were extracted and transesterified to fatty acid methyl esters, which were then separated and identified by gas-chromatography [45].

Determination of staphyloxanthin production in *S. aureus* strains grown in TSB, MHB and human serum. Staphyloxanthin content in the *S. aureus* cells was determined as described previously [23,46]. Briefly, TSB-, MHB- and serum-grown Pig1 cells were pelleted (3000× *g* for 5 min at 4 °C) at OD_600_ of ~0.8, and then at 12 h, 24 h and 48 h. Each pellet was washed with water and resuspended in 0.8 mL of 100% methanol and incubated at 65 °C for 10 min with vortexing every three minutes. OD_465_ was measured for each methanol extract and the staphyloxanthin-content was determined by dividing OD_465_ by cell mass (OD_600_ 1.0 corresponds to 0.39 mg dry wt./mL; Bieber and Wilkinson, 1984). Three independent assays were performed for each category.s

Determination of membrane fluidity of *S. aureus* strains grown in TSB, MHB and human serum. The membrane fluidities of the strains Pig1, Pig1Δ*crtM* and MHSR1132 were determined using 1,6-diphenyl-1,3,5-hexatriene (DPH, Sigma-Aldrich, St. Louis, MO, USA) as described previously [47] with some modifications. Briefly, the strains were grown in TSB, MHB and serum, pelleted, washed, resuspended in phosphate buffered saline (PBS, pH 7.5) to an OD_600_ of ~0.8, DPH (5 µM) was added and the suspension was incubated at 30 °C in a water bath. All steps involving DPH were carried out in the dark. Fluorescence polarization emitted by the fluorophore was measured using a PTIModel Quanta Master-4 Scanning Spectrofluorometer at an excitation wavelength of 360 nm and an emission wavelength of 430 nm. The experiments were performed with three separate fresh batch cultures of cells grown to early-exponential phase, 12, 24 and 48 h. 

Determination of expression of genes for staphyloxanthin and fatty acid biosynthesis and their regulation. The level of expression of the genes encoding key enzymes for staphyloxanthin and fatty acid biosynthesis and their regulation were determined by quantitative Real-time Polymerase Chain Reaction (qRT-PCR). Briefly, overnight grown cultures of the strains Pig1 and Pig1Δ*crtM* were inoculated in TSB or MHB, grown to OD_600_ of ~0.8, harvested, and total RNA was extracted and purified using a RNAeasy Mini Kit (Qiagen). cDNA was prepared from 300 ng of each RNA using the High Capacity RNA-to-cDNA kit (Applied Biosystems, Foster City, CA, USA). qRT-PCR reactions were conducted using the DyNAmo Flash SYGR Green Kit (Thermo Scientific, Waltham, MA, USA) with gene-specific primers (Supplementary Materials Table S1) along with for 16SrRNA as an internal control of gene expression. PCR was run using the ABI 7300 Real-Time PCR system. The levels of expression of the genes were calculated by the Comparative Ct method. The reactions were run in triplicates for each set and each experiment was repeated with three independent biological replicates. 

Determination of staphyloxanthin in Pig1 grown in glucose or acetate containing LB. Overnight grown culture was inoculated into (i) LB; (ii) LB with MOPS [3-(*N*-morpholino)propanesulfonic acid] buffer, pH 7.5; (iii) LB with 140 mM glucose; (iv) LB with MOPS and 140 mM glucose; and (v) LB with MOPS and 100 mM acetate. The cells were grown at 37 °C, harvested at 3 h and 12 h, and staphyloxanthin content was determined as described previously. Each experiment was repeated at least three times. 

Determination of acetate content in culture supernatants. Culture supernatants were collected from strain Pig1 grown in TSB, MHB, 140 mM glucose-supplemented TSB and MHB, and harvested (10,000× *g* for 10 min at 4 °C) at ~3 h (OD_600_ ~0.8) and at 12 h. The supernatants were filtered through 0.2 µ filter (Millipore) and acetate concentration was determined with an acetate standard calibration curve prepared using an Acetic Acid Assay Kit (Megazyme International Ireland, Wicklow, Ireland). Additionally, staphyloxanthin content in the cell pellets were determined. Each experiment was repeated three times.

## 5. Conclusions

Membrane biophysical properties are largely determined by the fatty acid composition of the cellular phospholipids. *S. aureus* produces BCFAs and SCSFAs and alters the balance of these fatty acids in response to environmental conditions. *S. aureus* also produces the hallmark membrane pigment, staphyloxanthin. Here we studied various aspects of fatty acid composition and pigmentation in pigmented and non-pigmented strains. We observed that the relationship of fatty acid composition and pigmentation in *S. aureus* is a complex phenomenon in which the availability of glucose in growth medium reduces both pigmentation and BCFAs while the availability of acetate increases pigmentation but decreases BCFAs. Our data suggests that the higher the production of BCFAs the higher there is acetate flux towards staphyloxanthin production through the mevalonate pathway. We believe that the overall relationship of fatty acid composition and pigmentation is largely regulated by carbon flow in *S. aureus*.

## Figures and Tables

**Table 1:** Fatty acid composition (%) ^1^ of strain Pig1 grown in various media.

Fatty Acids	Log ^2^			Stationary ^3^		
	TSB	MHB	Serum	TSB	MHB	Serum
SCSFAs	50.7 ± 0.28	19.0 ± 0.12	43.9 ± 0.25	24.1 ± 0.11	5.7 ± 0.03	44.0 ± 0.39
BCFAs	49.2 ± 0.20	80.9 ± 0.22	14.8 ± 0.18	75.9 ± 0.11	94.4 ± 0.04	19.9 ± 0.02
SCUFAs	nd	nd	41.4 ± 0.42	nd	nd	38.1 ± 0.40

^1^ Standard error of the mean (SEM) in the % of each fatty acid was determined from two independent fatty acid analysis experiments; ^2^ OD_600_ ~0.8; ^3^ 12 h. nd, not detected.

**Table 2:** Carotenoid content ^1^ (OD_465_/mg of dry cell mass) of Pig1 grown in various media.

Medium	Log ^2^	12 h	24 h	48 h
TSB	0.017 ± 0.0006	0.127 ± 0.0121	0.132 ± 0.0081	0.110 ± 0.0075
MHB	0.051 ± 0.0029	0.086 ± 0.0029	0.063 ± 0.0012	0.041 ± 0.0040
Serum	0.036 ± 0.0029	0.039 ± 0.0052	0.038 ± 0.0023	0.047 ± 0.0058

^1^ Standard error of the mean (SEM) in the carotenoid content was determined from at least three independent experiments; ^2^ OD_600_ ~0.8.

**Table 3:** Fatty acid composition (%) ^1^ of a carotenoid-deficient strain Pig1Δ*crtM* grown in various media.

Fatty Acids	Log ^2^			Stationary ^3^		
	TSB	MHB	Serum	TSB	MHB	Serum
SCSFAs	49.9 ± 0.06	19.6 ± 0.53	45.2 ± 0.07	24.5 ± 0.25	6.0 ± 0.03	45.6 ± 0.04
BCFAs	49.9 ± 0.13	80.4 ± 0.53	15.5 ± 0.33	75.5 ± 0.25	94.0 ± 0.02	18.1 ± 0.08
SCUFAs	nd	nd	39.3 ± 0.26	nd	nd	36.4 ± 0.01

^1^ Standard error of the mean (SEM) in the % of each fatty acid was determined from two independent fatty acid analysis experiments; ^2^ OD_600_ ~0.8; ^3^ 12 h. nd, not detected.

**Table 4:** Fatty acid composition (%) ^1^ of a naturally carotenoid-deficient strain MSHR1132 (*S. argenteus*) grown in various media.

Fatty Acids	Log ^2^			Stationary ^3^		
	TSB	MHB	Serum	TSB	MHB	Serum
SCSFAs	41.1 ± 0.86	17.5 ± 1.44	49.2 ± 0.19	20.9 ± 0.08	6.9 ± 0.04	49.8 ± 0.04
BCFAs	58.5 ± 1.05	82.2 ± 1.41	14.7 ± 0.04	79.1 ± 0.08	93.1 ± 0.03	12.9 ± 0.04
SCUFAs	nd	nd	36.2 ± 0.22	nd	nd	37.3 ± 0.01

^1^ Standard error of the mean (SEM) in the % of each fatty acid was determined from two independent fatty acid analysis experiments; ^2^ OD_600_ ~0.8; ^3^ 12 h. nd, not detected.

**Table 5:** Membrane anisotropy values ^1^ of *S. aureus* strains grown to log phase ^2^ in various media.

Strains	TSB	MHB	Serum
Pig1	0.315 ± 0.025	0.318 ± 0.035	0.212 ± 0.017
Pig1Δ*crtM*	0.260 ± 0.033	0.202 ± 0.032	0.159 ± 0.007
*S. argenteus*	0.254 ± 0.024	0.239 ± 0.010	0.135 ± 0.016

^1^ Standard error of the mean (SEM) in the anisotropy values was determined from at least three independent experiments. Statistical significance (Student’s paired t-test, two tail) values (*p*): Pig1_TSBvs.MHB_ 0.387; Pig1_TSBvs.Serum_ 0.0001; Pig1Δ*crtM*_TSBvs.MHB_ 0.003; Pig1Δ*crtM*_TSBvs.Serum_ 0.017; *S. argenteus*_TSBvs.MHB_ 0.189; *S. argenteus*_TSBvs.Serum_ 0.041; TSB_Pig1vs.Pig1Δ*crtM*_ 0.023; TSB_Pig1vs.*S.*_*_argenteus_* 0.023; ^2^ OD_600_ ~0.8.

**Table 6:** Staphyloxanthin content ^1^ (OD_465_/mg of dry cell mass) in Pig1 grown in various LB broths.

Growth Conditions	Log ^2^	Stationary ^3^
LB	0.03276 ± 0.0074	0.06030 ± 0.0072
LB-MOPS	0.03120 ± 0.0066	0.05814 ± 0.0073
LB-Glucose	0.02101 ± 0.0068	0.03297 ± 0.0042
LB-MOPS-Glucose	0.02040 ± 0.0061	0.03457 ± 0.0039
LB-MOPS-Acetate	0.03755 ± 0.0050	0.07133 ± 0.0040

^1^ Standard error of the mean (SEM) in the carotenoid content was determined from at least three independent experiments. Statistical significance (Student’s paired *t*-test, two tail) values (*p*): Log_LBvs.LB-MOPS_ 0.083; Log_LBvs.LB-Glucose_ 0.001; Log_LBvs.LB-MOPS-Glucose_ 0.001; Log_LB-Glucosevs.LB-MOPS-Glucose_ 0.455; Log_LBvs.LB-MOPS-Acetate_ 0.109; Stationary_LBvs.LB-MOPS_ 0.221; Stationary_LBvs.LB-Glucose_ 0.0007; Stationary_LBvs.LB-MOPS-Glucose_ 0.001; Stationary_LB-Glucosevs.LB-MOPS-Glucose_ 0.146; Stationary_LBvs.LB-MOPS-Acetate_ 0.019; ^2^ OD_600_ ~0.8. ^3^ 12 h.

**Table 7:** Fatty acid composition (%) ^1^ in *S. aureus* Pig1 grown at stationary phase ^2^ in glucose or acetate containing LB broths.

Fatty Acids	LB	LB-MOPS-Glucose	LB-MOPS-Acetate
SCSFA	9.2 ± 0.30	38.0 ± 1.00	27.1 ± 0.04
BCFA	90.8 ± 0.28	61.9 ± 0.95	62.8 ± 0.15
Unknown	nd	nd	10.2 ± 0.11

^1^ Standard error of the mean (SEM) in the % of each fatty acid was determined from two independent fatty acid analysis experiments; ^2^ Fatty acid analysis was done for the stationary phase (12 h grown) cells that had significant differences in pigment production (see Table 6). nd, not detected.

**Table 8:** Medium acetate ^1^ concentration and staphyloxanthin production in *S. aureus* strain Pig1.

	[Acetate] ^2^, mM		Staphyloxanthin ^3^	
	Log	Stationary	Log	Stationary
TSB	8.50 ± 0.99	19.38 ± 2.31	0.017 ± 0.0006	0.127 ± 0.0121
TSB-Glucose	8.07 ± 1.42	25.82 ± 1.85	0.018 ± 0.0023	0.033 ± 0.0012
MHB	3.09 ± 0.64	7.33 ± 1.02	0.051 ± 0.0029	0.086 ± 0.0029
MHB-Glucose	5.17 ± 0.98	27.07 ± 1.63	0.035 ± 0.0006	0.031 ± 0.0046

^1^ Standard error of the mean (SEM) in the acetate concentration was determined from three independent experiments; ^2^ Acetate concentration was determined in the supernatants of log (OD_600_ ~0.8) and stationary phase (12 h; TSB, OD_600_ ~8.0; MHB, OD_600_ ~6.0) cultures; ^3^ OD_465_ per mg dry weight of cell mass.

**Table 9:** Description of the strains used in this study.

Strains	Description	References
*S. aureus* Pig1	Markedly pigmented clinical isolate	[14]
*S. aureus* Pig1Δ*crtM*	*crtM*-knockout construct of *S. aureus* Pig1	[14]
*S. argenteus*	Naturally *crt*-operon lacking clinical strain. Closely related to *S. aureus*. Also referred to as *S. aureus* MSHR1132	[19,20]
*S. aureus* SH1000	A laboratory-derived *S. aureus* strain NCTC 8325-4 by complementing *rsbU*^+^ from *S. aureus* Newman	[41]
*S. aureus* Newman	Clinical isolate sensitive to methicillin	[42]
*S. aureus* COL	Hospital-associated homogeneous methicillin-resistant strain	[43]
*S. aureus* MW2	Community-associated heterogeneous methicillin-resistant strain	[44]

## Supplementary Materials

The following are available online, Table S1: Primer sequences used in qRT-PCR assays. Table S2: Fatty acid profiles of *S. aureus* grown in various media.
