# Supplementary material for: Interrelationships among Fatty Acid Composition, Staphyloxanthin Content, Fluidity, and Carbon Flow in the Staphylococcus aureus Membrane
Source: Molecules. 2018 May 17;23(5):1201. doi: 10.3390/molecules23051201 (PMC6099573; doi:10.3390/molecules23051201)
Supplement: Supplementary file 1 [file molecules-23-01201-s001.zip › molecules-295350-supplementary-revised/molecules-295350-supplementary -table S1.pdf]

Table S1. Sequences of the primers used in qRT-PCR assay

Primer	Sequence
crtM-F	CAATGTTTGAAACGGACGCTG
crtM-R	CGATTCACCAAGTCTTCTTGCG
rsbV-F	TATGGATTCGACAGGTTTAGGT
rsbV-R	ACCGATACGATCTGACACAC
sigB-F	TCTGATCGCGAACGAGAAATC
sigB-R	ATTGCCGTTCTCTGAAGTCGT
fabH-F	TAAAAGCAATCGCTGACGCTG
fabH-R	CAACTTTGCCCGTCCCTAAAC
lpd-F	TGCAGCTGGTGATTGTATTGG
lpd-R	TACTGGGATTGGGTTCCCCT
fakA-F	GCAAGTGAACAAGCAGCGAG
fakA-R	TTGCGTCCACATCACATTGG
fapR-F	AAAACTGGAATTGCGCGTGG
fapR-R	TCGTGCTTCTGCTCTTACCG
16SrRNA-F	CTGGAACTGAGACACGGTCC
16SrRNA-R	GACCTTCATCACTCACGCGG
