# Supplementary material for: Interrelationships among Fatty Acid Composition, Staphyloxanthin Content, Fluidity, and Carbon Flow in the Staphylococcus aureus Membrane
Source: Molecules. 2018 May 17;23(5):1201. doi: 10.3390/molecules23051201 (PMC6099573; doi:10.3390/molecules23051201)
Supplement: Supplementary file 1 [file molecules-23-01201-s001.zip › Table S1. Sequences of the primers used in qRT-PCR assay.docx]

Table S1. Sequences of the primers used in qRT-PCR assay

| **Primer** | **Sequence** |
| --- | --- |
| *crtM*-F | CAATGTTTGAAACGGACGCTG |
| *crtM*-R | CGATTCACCAAGTCTTCTTGCG |
| *rsbV*-F | TATGGATTCGACAGGTTTAGGT |
| *rsbV*-R | ACCGATACGATCTGACACAC |
| *sigB*-F | TCTGATCGCGAACGAGAAATC |
| *sigB*-R | ATTGCCGTTCTCTGAAGTCGT |
| *fabH*-F | TAAAAGCAATCGCTGACGCTG |
| *fabH*-R | CAACTTTGCCCGTCCCTAAAC |
| *lpd*-F | TGCAGCTGGTGATTGTATTGG |
| *lpd*-R | TACTGGGATTGGGTTCCCCT |
| *fakA*-F | GCAAGTGAACAAGCAGCGAG |
| *fakA*-R | TTGCGTCCACATCACATTGG |
| *fapR*-F | AAAACTGGAATTGCGCGTGG |
| *fapR*-R | TCGTGCTTCTGCTCTTACCG |
| *16SrRNA*-F | CTGGAACTGAGACACGGTCC |
| *16SrRNA*-R | GACCTTCATCACTCACGCGG |
